# Supplementary material for: Cortical adaptation to sound reverberation
Source: eLife. 2022 May 26;11:e75090. doi: 10.7554/eLife.75090 (PMC9213001; doi:10.7554/eLife.75090)
Supplement: Supplementary file 1. [file elife-75090-supp1.docx]

**Supplementary File 1. Supplementary statistics for Wilcoxon signed-rank and** **Kruskal-Wallis tests**

#

# 1. Figure 3A

Paired Wilcoxon signed-rank tests comparing the model kernel centre of mass (COM) values.

| Group | Median (ms) | Standard deviation (ms) | N | Z value | p value | Effect size  (Rosenthal’s correlation r) |
| --- | --- | --- | --- | --- | --- | --- |
| Inhibitory COM large room | 66.0 | 4.6 | 30 | 4.8 | 1.9x10^-6^ | 0.87 |
| Inhibitory COM small room | 59.0 | 3.5 |  |  |  |  |
| Excitatory COM large room | 40.0 | 4.2 | 30 | 1.8 | 0.066 | 0.37 |
| Excitatory COM small room | 38.0 | 4.0 |  |  |  |  |

# 2. Figure 3B

Paired Wilcoxon signed-rank tests comparing the ferret auditory cortical neurons STRF COM values.

| Group | Median (ms) | Standard deviation (ms) | N | Z value | p value | Effect size  (Rosenthal’s correlation r) |
| --- | --- | --- | --- | --- | --- | --- |
| Inhibitory COM large room | 85.0 | 25.0 | 696 | 17.0 | 1.5x10^-66^ | 0.65 |
| Inhibitory COM small room | 74.0 | 19.0 |  |  |  |  |
| Excitatory COM large room | 44.0 | 25.0 | 696 | 0.87 | 0.39 | 0.033 |
| Excitatory COM small room | 44.0 | 22.0 |  |  |  |  |

**3. Figure 3C**

Paired Wilcoxon signed-rank tests comparing the model kernels’ peak time (PT) values.

| Group | Median (ms) | Standard deviation (ms) | N | Z value | p value | Effect size  (Rosenthal’s correlation r) |
| --- | --- | --- | --- | --- | --- | --- |
| Inhibitory PT large room | 14.0 | 17.0 | 30 | 2.9 | 0.0037 | 0.53 |
| Inhibitory PT small room | 9.2 | 3.8 |  |  |  |  |
| Excitatory PT large room | 0.0 | 0 | 30 | 0.0 | 1.0 | 0.0 |
| Excitatory PT small room | 0.0 | 0 |  |  |  |  |

# 4. Figure 3D

Paired Wilcoxon signed-rank tests comparing the ferret auditory cortical neurons STRF PT values.

| Group | Median (ms) | Standard deviation (ms) | N | Z value | p value | Effect size  (Rosenthal’s correlation r) |
| --- | --- | --- | --- | --- | --- | --- |
| Inhibitory PT large room | 69.0 | 48.0 | 696 | 14.0 | 4.0x10^-44^ | 0.53 |
| Inhibitory PT small room | 50.0 | 33.0 |  |  |  |  |
| Excitatory PT large room | 16.0 | 36.0 | 696 | 0.89 | 0.38 | 0.034 |
| Excitatory PT small room | 16.0 | 33.0 |  |  |  |  |

# 5. Figure 3-Figure supplement 1A

Kruskal-Wallis comparing ferret auditory cortical neurons STRF COM excitatory values with room size (large, medium, small) as a predictor.

| Dependent variable | Degrees of freedom | 𝛘^2^ value | p value | Effect size (Eta squared) |
| --- | --- | --- | --- | --- |
| Room size | 2 | 6.4 | 0.042 | 0.008 |

Post-hoc pairwise comparisons between COM excitatory values of rooms of different sizes performed on the results of the above Kruskal-Wallis, using Fisher’s least significant difference (LSD) procedure.

| Compared room sizes | | Lower confidence interval (ms) | Median (ms) | Upper confidence interval (ms) | p value |
| --- | --- | --- | --- | --- | --- |
| Medium room | Small room | 11.0 | 50.0 | 89.0 | 0.0117 |
| Large room | Small room | -14.0 | 25.0 | 64.0 | 0.21 |
| Large room | Medium room | -64.0 | -25.0 | 14.0 | 0.21 |

# 6. Figure 3-Figure supplement 1B

Kruskal-Wallis comparing ferret auditory cortical neurons STRF COM inhibitory values with room size (large, medium, small) as a predictor.

| Dependent variable | Degrees of freedom | 𝛘^2^ value | p value | Effect size (Eta squared) |
| --- | --- | --- | --- | --- |
| Room size | 2 | 37.0 | 7.6x10^-9^ | 0.047 |

Post-hoc pairwise comparisons between COM inhibitory values of rooms of different sizes performed on the results of the above Kruskal-Wallis, using Fisher’s least significant difference (LSD) procedure.

| Compared room sizes | | Lower confidence interval (ms) | Median (ms) | Upper confidence interval (ms) | p value |
| --- | --- | --- | --- | --- | --- |
| Medium room | Small room | 7.7 | 47.0 | 86.0 | 0.0189 |
| Large room | Small room | 82.0 | 121.0 | 160.0 | 1.3x10^-9^ |
| Large room | Medium room | 35.0 | 74.0 | 113.0 | 2.0x10^-4^ |

# 7. Figure 3-Figure supplement 1C

Kruskal-Wallis comparing ferret auditory cortical neurons STRF PT excitatory values with room size (large, medium, small) as a predictor.

| Dependent variable | Degrees of freedom | 𝛘^2^ value | p value | Effect size (Eta squared) |
| --- | --- | --- | --- | --- |
| Room size | 2 | 1.4 | 0.50 | 0.0017 |

Post-hoc pairwise comparisons between PT excitatory values of rooms of different sizes performed on the results of the above Kruskal-Wallis, using Fisher’s least significant difference (LSD) procedure.

| Compared room sizes | | Lower confidence interval (ms) | Median (ms) | Upper confidence interval (ms) | p value |
| --- | --- | --- | --- | --- | --- |
| Medium room | Small room | -36.0 | -1.6 | 33.0 | 0.93 |
| Large room | Small room | -18.0 | 17.0 | 52.0 | 0.33 |
| Large room | Medium room | -16.0 | 19.0 | 54.0 | 0.29 |

# 8. Figure 3-Figure supplement 1D

Kruskal-Wallis comparing ferret auditory cortical neurons STRF PT inhibitory values with room size (large, medium, small) as a predictor.

| Dependent variable | Degrees of freedom | 𝛘^2^ value | p value | Effect size (Eta squared) |
| --- | --- | --- | --- | --- |
| Room size | 2 | 27.0 | 1.6x10^-6^ | 0.034 |

Post-hoc pairwise comparisons between PT inhibitory values of rooms of different sizes performed on the results of the above Kruskal-Wallis, using Fisher’s least significant difference (LSD) procedure.

| Compared room sizes | | Lower confidence interval (ms) | Median (ms) | Upper confidence interval (ms) | p value |
| --- | --- | --- | --- | --- | --- |
| Medium room | Small room | 2.8 | 41.0 | 80.0 | 0.036 |
| Large room | Small room | 63.0 | 101.0 | 140.0 | 2.7x10^-7^ |
| Large room | Medium room | 21.0 | 60.0 | 98.0 | 0.0024 |

# 9. Figure 3-Figure supplement 2E

Paired Wilcoxon signed-rank tests comparing the Bruce Erfani Zilany (BEZ) cochleagram model kernel centre of mass (COM) values.

| Group | Median (ms) | Standard deviation (ms) | N | Z value | p value | Effect size  (Rosenthal’s correlation r) |
| --- | --- | --- | --- | --- | --- | --- |
| Inhibitory COM large room | 78.0 | 6.8 | 30 | 4.8 | 1.7x10^-6^ | 0.87 |
| Inhibitory COM small room | 68.0 | 4.6 |  |  |  |  |
| Excitatory COM large room | 34.0 | 8.7 | 30 | -4.3 | 1.6x10^-5^ | -0.79 |
| Excitatory COM small room | 40.0 | 7.2 |  |  |  |  |

#

# 10. Figure 3-Figure supplement 2F

Paired Wilcoxon signed-rank tests comparing the BEZ cochleagram ferret auditory cortical neurons STRF COM values.

| Group | Median (ms) | Standard deviation (ms) | N | Z value | p value | Effect size  (Rosenthal’s correlation r) |
| --- | --- | --- | --- | --- | --- | --- |
| Inhibitory COM large room | 96.0 | 24.0 | 696 | 19.0 | 4.2x10^-78^ | 0.71 |
| Inhibitory COM small room | 83.0 | 19.0 |  |  |  |  |
| Excitatory COM large room | 41.0 | 26.0 | 696 | 5.3 | 1.2x10^-7^ | 0.20 |
| Excitatory COM small room | 38.0 | 25.0 |  |  |  |  |

# 11. Figure 3-Figure supplement 2G

Paired Wilcoxon signed-rank tests comparing the BEZ cochleagram model kernel peak time (PT) values.

| Group | Median (ms) | Standard deviation (ms) | N | Z value | p value | Effect size  (Rosenthal’s correlation r) |
| --- | --- | --- | --- | --- | --- | --- |
| Inhibitory PT large room | 30.0 | 33.0 | 30 | 4.8 | 4.7x10^-6^ | 0.84 |
| Inhibitory PT small room | 9.2 | 0.48 |  |  |  |  |
| Excitatory PT large room | 0.0 | 0 | 30 | 0.0 | 1.0 | 0.0 |
| Excitatory PT small room | 0.0 | 0 |  |  |  |  |

# 12. Figure 3-Figure supplement 2H

Paired Wilcoxon signed-rank tests comparing the BEZ cochleagram ferret auditory cortical neurons STRF PT values.

| Group | Median (ms) | Standard deviation (ms) | N | Z value | p value | Effect size  (Rosenthal’s correlation r) |
| --- | --- | --- | --- | --- | --- | --- |
| Inhibitory PT large room | 80.0 | 50.0 | 696 | 16.0 | 4.6x10^-58^ | 0.61 |
| Inhibitory PT small room | 57.0 | 36.0 |  |  |  |  |
| Excitatory PT large room | 19.0 | 38.0 | 696 | 5.8 | 6.2x10^-9^ | 0.22 |
| Excitatory PT small room | 18.0 | 37.0 |  |  |  |  |

#

# 13. Figure 4B

Paired Wilcoxon signed-rank tests comparing the LNP simulated model neurons STRF COM values.

| Group | Median (ms) | Standard deviation (ms) | N | Z value | p value | Effect size  (Rosenthal’s correlation r) |
| --- | --- | --- | --- | --- | --- | --- |
| Inhibitory COM large room | 83.0 | 21.0 | 696 | 14.0 | 9.5x10^-42^ | 0.51 |
| Inhibitory COM small room | 78.0 | 19.0 |  |  |  |  |
| Excitatory COM large room | 53.0 | 22.0 | 696 | 6.8 | 9.0x10^-12^ | 0.26 |
| Excitatory COM small room | 49.0 | 22.0 |  |  |  |  |

# 14. Figure 4C

Paired Wilcoxon signed-rank tests comparing the real ferret auditory cortical STRF COM values.

| Group | Median (ms) | Standard deviation (ms) | N | Z value | p value | Effect size  (Rosenthal’s correlation r) |
| --- | --- | --- | --- | --- | --- | --- |
| Inhibitory COM large room | 85.0 | 25.0 | 696 | 17.0 | 1.5x10^-66^ | 0.65 |
| Inhibitory COM small room | 74.0 | 19.0 |  |  |  |  |
| Excitatory COM large room | 44.0 | 25.0 | 696 | 0.87 | 0.39 | 0.033 |
| Excitatory COM small room | 44.0 | 22.0 |  |  |  |  |

#

# 15. Figure 4D

Paired Wilcoxon signed-rank tests comparing the LNP simulated model neurons with real ferret auditory cortical neurons STRF COM values.

| Group | Median (ms) | Standard deviation (ms) | N | Z value | p value | Effect size  (Rosenthal’s correlation r) |
| --- | --- | --- | --- | --- | --- | --- |
| Inhibitory COM difference in real neurons | 9.3 | 17.0 | 696 | 11.0 | 7.2x10^-29^ | 0.42 |
| Inhibitory COM difference in simulated neurons | 4.0 | 11.0 |  |  |  |  |
| Excitatory COM difference in real neurons | 0.32 | 17.0 | 696 | -3.5 | 4.9x10^-4^ | -0.13 |
| Excitatory COM difference in simulated neurons | 3.1 | 13.0 |  |  |  |  |

# 16. Figure 4E

Paired Wilcoxon signed-rank tests comparing the LNP simulated model neurons STRF PT values.

| Group | Median (ms) | Standard deviation (ms) | N | Z value | p value | Effect size  (Rosenthal’s correlation r) |
| --- | --- | --- | --- | --- | --- | --- |
| Inhibitory PT large room | 80.0 | 43.0 | 696 | 12.0 | 5.9x10^-32^ | 0.45 |
| Inhibitory PT small room | 60.0 | 38.0 |  |  |  |  |
| Excitatory PT large room | 9.6 | 37.0 | 696 | -5.4 | 6.2x10^-8^ | -0.21 |
| Excitatory PT small room | 15.0 | 36.0 |  |  |  |  |

#

# 17. Figure 4F

Paired Wilcoxon signed-rank tests comparing the real ferret auditory cortical neurons STRF peak time PT values.

| Group | Median (ms) | Standard deviation (ms) | N | Z value | p value | Effect size  (Rosenthal’s correlation r) |
| --- | --- | --- | --- | --- | --- | --- |
| Inhibitory PT large room | 69.0 | 48.0 | 696 | 14.0 | 4.0x10^-44^ | 0.53 |
| Inhibitory PT small room | 50.0 | 33.0 |  |  |  |  |
| Excitatory PT large room | 16.0 | 36.0 | 696 | 0.89 | 0.38 | 0.034 |
| Excitatory PT small room | 16.0 | 33.0 |  |  |  |  |

#

# 18. Figure 4G

Paired Wilcoxon signed-rank tests comparing the LNP simulated model neurons with real ferret auditory cortical neurons STRF PT values.

| Group | Median (ms) | Standard deviation (ms) | N | Z value | p value | Effect size  (Rosenthal’s correlation r) |
| --- | --- | --- | --- | --- | --- | --- |
| Inhibitory PT difference in real neurons | 9.4 | 42.0 | 696 | 3.6 | 3.6x10^-4^ | 0.14 |
| Inhibitory PT difference in simulated neurons | 6.4 | 35.0 |  |  |  |  |
| Excitatory PT difference in real neurons | 0.0 | 30.0 | 696 | 5.4 | 6.0x10^-8^ | 0.21 |
| Excitatory PT difference in simulated neurons | -0.5 | 28.0 |  |  |  |  |

# 19. Figure 4-Figure supplement 1A

Paired Wilcoxon signed-rank tests comparing the Network Receptive Field-Poisson (NRFP) simulated model neurons STRF COM values.

| Group | Median (ms) | Standard deviation (ms) | N | Z value | p value | Effect size  (Rosenthal’s correlation r) |
| --- | --- | --- | --- | --- | --- | --- |
| Inhibitory COM large room | 85.0 | 19.0 | 696 | 14.0 | 2.6x10^-46^ | 0.54 |
| Inhibitory COM small room | 80.0 | 18.0 |  |  |  |  |
| Excitatory COM large room | 60.0 | 23.0 | 696 | 7.8 | 5.9x10^-15^ | 0.30 |
| Excitatory COM small room | 57.0 | 23.0 |  |  |  |  |

# 20. Figure 4-Figure supplement 1B

Paired Wilcoxon signed-rank tests comparing the NRFP simulated model neurons with real ferret auditory cortical neurons STRF COM values.

| Group | Median (ms) | Standard deviation (ms) | N | Z value | p value | Effect size  (Rosenthal’s correlation r) |
| --- | --- | --- | --- | --- | --- | --- |
| Inhibitory COM difference in real neurons | 9.3 | 17.0 | 696 | 9.8 | 7.0x10^-23^ | 0.37 |
| Inhibitory COM difference in simulated neurons | 5.6 | 12.0 |  |  |  |  |
| Excitatory COM difference in real neurons | 0.32 | 17.0 | 696 | -4.0 | 8.0x10^-5^ | -0.15 |
| Excitatory COM difference in simulated neurons | 3.5 | 13.0 |  |  |  |  |

#

#

#

# 21. Figure 4-Figure supplement 1C

Paired Wilcoxon signed-rank tests comparing the NRFP simulated model neurons STRF PT values.

| Group | Median (ms) | Standard deviation (ms) | N | Z value | p value | Effect size  (Rosenthal’s correlation r) |
| --- | --- | --- | --- | --- | --- | --- |
| Inhibitory PT large room | 60.0 | 46.0 | 696 | 7.8 | 6.6x10^-15^ | 0.30 |
| Inhibitory PT small room | 51.0 | 38.0 |  |  |  |  |
| Excitatory PT large room | 11.0 | 40.0 | 696 | -5.2 | 2.3x10^-7^ | -0.20 |
| Excitatory PT small room | 17.0 | 36.0 |  |  |  |  |

# 22. Figure 4-Figure supplement 1D

Paired Wilcoxon signed-rank tests comparing the NRFP simulated model neurons with real ferret auditory cortical neurons STRF PT values.

| Group | Median (ms) | Standard deviation (ms) | N | Z value | p value | Effect size  (Rosenthal’s correlation r) |
| --- | --- | --- | --- | --- | --- | --- |
| Inhibitory PT difference in real neurons | 9.4 | 42.0 | 696 | 5.2 | 2.5x10^-7^ | 0.20 |
| Inhibitory PT difference in simulated neurons | 2.1 | 44.0 |  |  |  |  |
| Excitatory PT difference in real neurons | 0 | 30.0 | 696 | 4.3 | 1.6x10^-5^ | 0.16 |
| Excitatory PT difference in simulated neurons | -0.4 | 33.0 |  |  |  |  |

#

# 23. Figure 5B

Paired Wilcoxon signed-rank test comparing the ferret auditory cortical neurons responses COM to a noise probe stimulus.

| Group | Median (ms) | Standard deviation (ms) | N | Z value | p value | Effect size  (Rosenthal’s correlation r) |
| --- | --- | --- | --- | --- | --- | --- |
| Noise probe COM large room | 40.0 | 14.0 | 289 | 2.7 | 0.0063 | 0.16 |
| Noise probe COM small room | 36.0 | 15.0 |  |  |  |  |

# 24. Figure 5D

Paired Wilcoxon signed-rank tests comparing the ferret auditory cortical neurons STRF COM values in response to switching stimuli in the small room condition.

| Group | Median (ms) | Standard deviation (ms) | N | Z value | p value | Effect size  (Rosenthal’s correlation r) |
| --- | --- | --- | --- | --- | --- | --- |
| Inhibitory COM S2 period | 83.0 | 20.0 | 327 | 2.6 | 0.0088 | 0.14 |
| Inhibitory COM S1 period | 85.0 | 20.0 |  |  |  |  |
| Excitatory COM S2 period | 48.0 | 18.0 | 327 | 0.93 | 0.35 | 0.052 |
| Excitatory COM S1 period | 46.0 | 16.0 |  |  |  |  |

# 25. Figure 5E

Paired Wilcoxon signed-rank tests comparing the ferret auditory cortical neurons STRF COM values in response to switching stimuli in the large room condition.

| Group | Median (ms) | Standard deviation (ms) | N | Z value | p value | Effect size  (Rosenthal’s correlation r) |
| --- | --- | --- | --- | --- | --- | --- |
| Inhibitory COM L2 period | 100.0 | 27.0 | 327 | -2.3 | 0.019 | -0.13 |
| Inhibitory COM L1 period | 96.0 | 27.0 |  |  |  |  |
| Excitatory COM L2 period | 49.0 | 21.0 | 327 | 0.19 | 0.85 | 0.010 |
| Excitatory COM L1 period | 48.0 | 22.0 |  |  |  |  |

# 26. Figure 6A

Paired Wilcoxon signed-rank tests comparing the Pearson’s correlation of ferret auditory cortical neurons spike train correlations to that of a non-adapting Linear-Nonlinear-Poisson (LNP) model in the small and anechoic room conditions.

| Group | Median | Standard deviation | N | Z value | p value | Effect size  (Rosenthal’s correlation r) |
| --- | --- | --- | --- | --- | --- | --- |
| Pearson’s correlation coefficient between the spike trains of the small and anechoic rooms in real neurons | 0.12 | 0.091 | 430 | 6.0 | 1.5x10^-9^ | 0.29 |
| Pearson’s correlation coefficient between the spike trains of the small and anechoic rooms in LNP model | 0.096 | 0.096 |  |  |  |  |

# 27. Figure 6B

Paired Wilcoxon signed-rank tests comparing the Pearson’s correlation of ferret auditory cortical neurons spike train correlations to that of a non-adapting Linear-Nonlinear-Poisson (LNP) model in the large and anechoic room conditions.

| Group | Median | Standard deviation | N | Z value | p value | Effect size  (Rosenthal’s correlation r) |
| --- | --- | --- | --- | --- | --- | --- |
| Pearson’s correlation coefficient between the spike trains of the large and anechoic rooms in real neurons | 0.061 | 0.071 | 430 | 6.9 | 7.2x10^-12^ | 0.33 |
| Pearson’s correlation coefficient between the spike trains of the large and anechoic rooms in LNP model | 0.048 | 0.053 |  |  |  |  |

#

# 28. Figure 6C

Paired Wilcoxon signed-rank tests comparing the Pearson’s correlation of ferret auditory cortical neurons spike train correlations to that of a non-adapting Linear-Nonlinear-Poisson (LNP) model in the large and small room conditions.

| Group | Median | Standard deviation | N | Z value | p value | Effect size  (Rosenthal’s correlation r) |
| --- | --- | --- | --- | --- | --- | --- |
| Pearson’s correlation coefficient between the spike trains of the large and small rooms in real neurons | 0.11 | 0.092 | 430 | 13.0 | 1.0x10^-40^ | 0.64 |
| Pearson’s correlation coefficient between the spike trains of the large and small rooms in LNP model | 0.068 | 0.075 |  |  |  |  |

# 29. Figure 6-Figure supplement 1A

Paired Wilcoxon signed-rank tests comparing the Pearson’s correlation of dereverberation model predicted anechoic cochleagram from the small original cochleagram with the original anechoic cochleagram to that of the original small and anechoic cochleagrams, across frequency channels.

| Group | Median | Standard deviation | N | Z value | p value | Effect size  (Rosenthal’s correlation r) |
| --- | --- | --- | --- | --- | --- | --- |
| Pearson’s correlation coefficient between the predicted anechoic cochleagram from the small cochleagram with the original anechoic cochleagram across frequency channels | 0.88 | 0.0062 | 30 | 4.8 | 1.7x10^-6^ | 0.87 |
| Pearson’s correlation coefficient between the original small and anechoic cochleagrams across frequency channels | 0.82 | 0.022 |  |  |  |  |

# 30. Figure 6-Figure supplement 1B

Paired Wilcoxon signed-rank tests comparing the Pearson’s correlation of dereverberation model predicted anechoic cochleagram from the large original cochleagram with the original anechoic cochleagram to that of the original large and anechoic cochleagrams, across frequency channels.

| Group | Median | Standard deviation | N | Z value | p value | Effect size  (Rosenthal’s correlation r) |
| --- | --- | --- | --- | --- | --- | --- |
| Pearson’s correlation coefficient between the predicted anechoic cochleagram from the large cochleagram with the original anechoic cochleagram across frequency channels | 0.83 | 0.022 | 30 | 4.8 | 1.7x10^-6^ | 0.87 |
| Pearson’s correlation coefficient between the original large and anechoic cochleagrams across frequency channels | 0.73 | 0.054 |  |  |  |  |

#

# 31. Figure 6-Figure supplement 1C

Paired Wilcoxon signed-rank tests comparing the Pearson’s correlation of dereverberation model predicted anechoic cochleagram from the large and small original cochleagrams to that of the original large and small cochleagrams, across frequency channels.

| Group | Median | Standard deviation | N | Z value | p value | Effect size  (Rosenthal’s correlation r) |
| --- | --- | --- | --- | --- | --- | --- |
| Pearson’s correlation coefficient between the predicted anechoic cochleagram from the large and small original cochleagrams across frequency channels | 0.91 | 0.012 | 30 | 4.8 | 1.7x10^-53^ | 0.87 |
| Pearson’s correlation coefficient between the original large and small cochleagrams across frequency channels | 0.88 | 0.019 |  |  |  |  |
