# Supplementary material for: Cortical adaptation to sound reverberation
Source: eLife. 2022 May 26;11:e75090. doi: 10.7554/eLife.75090 (PMC9213001; doi:10.7554/eLife.75090)
Supplement: Transparent reporting form [file elife-75090-transrepform1.pdf]

eLIFE
1st Floor
24 Hills Road
Cambridge CB2 1JP, UK
P 01223 855340
W elifesciences.org
T @elife

eLife's transparent reporting form

We encourage authors to provide detailed information *within their submission* to facilitate the interpretation and replication of experiments. Authors can upload supporting documentation to indicate the use of appropriate reporting guidelines for health-related research (see EQUATOR Network), life science research (see the BioSharing Information Resource), or the ARRIVE guidelines for reporting work involving animal research. Where applicable, authors should refer to any relevant reporting standards documents in this form.

If you have any questions, please consult our Journal Policies and/or contact us: editorial@elifesciences.org.

Sample-size estimation

- You should state whether an appropriate sample size was computed when the study was being designed
- You should state the statistical method of sample size computation and any required assumptions
- If no explicit power analysis was used, you should describe how you decided what sample (replicate) size (number) to use

Please outline where this information can be found within the submission (e.g., sections or figure legends), or explain why this information doesn't apply to your submission:

Our sample size was determined based on a rich literature of previous studies employing electrophysiological microelectrode recordings to examine auditory cortical receptive fields and their adaptive properties. The use of high-channel-count microelectrodes allowed us to reduce the number of animals required to get an adequate sample of neurons. The study required measuring neurons with best frequencies across the entire low-high frequency range, so that we could examine the frequency dependence of adaptation to reverberation. Based on our previous experience with these analyses, we aimed for at least 20 cortical units per frequency band tested. Including 7 ferrets allowed us to determine that our results were generalizable across individual animals.

The sample sizes and other relevant statistics are reported for each statistical test, either in the text of the paper or in the supplementary statistics tables.

Replicates

- You should report how often each experiment was performed
- You should include a definition of biological versus technical replication
- The data obtained should be provided and sufficient information should be provided to indicate the number of independent biological and/or technical replicates
- If you encountered any outliers, you should describe how these were handled
- Criteria for exclusion/inclusion of data should be clearly stated
- High-throughput sequence data should be uploaded before submission, with a private link for reviewers provided (these are available from both GEO and ArrayExpress)

Please outline where this information can be found within the submission (e.g., sections or figure legends), or explain why this information doesn't apply to your submission:

For all the datasets used, the number of replications is given in the manuscript text. In the case of the datasets we used, a biological replicate is a cortical unit, which is either a single-unit (i.e. an individual neuron) or multi-unit cluster (i.e. a collection of nearby neurons recorded at one electrode site) whose response to a stimulus is recorded. A technical replicate is a repeat of the same stimulus played to the cell. We make it clear in the manuscript where one or the other is used, denoting biological replicates by 'cortical units' and technical replicates by 'repeats', and we give the number of replicates used where relevant. In the Methods section ("Spike sorting" on page 16), we state very explicitly the criteria used for data exclusion and indicate the number of neural units included and excluded in each analysis. No data were excluded simply on the grounds of being an outlier.

Specifically, most analyses used 696 units from 7 ferrets, and this is stated in the Results (section "Auditory cortical neurons…" on pg. 4), and the Methods and Materials (sections "Animals" and "Spike sorting" on pg. 16). The exceptions to this are the results in Figure 3 Supplement 1 which used 266 units from 2 ferrets (stated in Results section "Similar effects…" on pg. 8) and the results in Figure 3 Supplement 4 which used 310 units from 4 ferrets (state in Results section "Reverberation effects…" on pg. 9). In all cases the number of repeats of each given stimulus is 10, and this is stated in the Methods and Materials (section "Sound stimuli…" on pg. 17; and section "Switching stimuli…" on pg. 22).

Statistical reporting

- Statistical analysis methods should be described and justified
- Raw data should be presented in figures whenever informative to do so (typically when N per group is less than 10)
- For each experiment, you should identify the statistical tests used, exact values of N, definitions of center, methods of multiple test correction, and dispersion and precision measures (e.g., mean, median, SD, SEM, confidence intervals; and, for the major substantive results, a measure of effect size (e.g., Pearson's r, Cohen's d)
- Report exact p-values wherever possible alongside the summary statistics and 95% confidence intervals. These should be reported for all key questions and not only when the p-value is less than 0.05.

Please outline where this information can be found within the submission (e.g., sections or figure legends), or explain why this information doesn't apply to your submission:

The values for statistical tests and analyses are found throughout the Results section of the manuscript, with extensive details of all statistical tests (e.g. effect sizes) provided in the supplementary statistical tables document. Exact p-values are given throughout the manuscript and in the tables.

We present the data at the most informative level for the questions we ask, which in this case is the receptive field, which also allows us to directly compare the neuronal data to the computational model.

We show example model kernels and neuronal receptive fields in Figure 2, and we also show averaged model kernels and receptive fields at each frequency channel within our examined range (Figure 2-Figure supplements 1,2).

eLife Sciences Publications, Ltd is a limited liability non-profit non-stock corporation incorporated in the State of Delaware, USA, with company number 5030732, and is registered in the UK with company number FC030576 and branch number BR015634 at the address 1st Floor, 24 Hills Road, Cambridge CB2 1JP | August 2014

(For large datasets, or papers with a very large number of statistical tests, you may upload a single table file with tests, Ns, etc., with reference to sections in the manuscript.)

Group allocation

- Indicate how samples were allocated into experimental groups (in the case of clinical studies, please specify allocation to treatment method); if randomization was used, please also state if restricted randomization was applied
- Indicate if masking was used during group allocation, data collection and/or data analysis

Please outline where this information can be found within the submission (e.g., sections or figure legends), or explain why this information doesn't apply to your submission:

Not applicable to our experimental design.

Additional data files ("source data")

- We encourage you to upload relevant additional data files, such as numerical data that are represented as a graph in a figure, or as a summary table
- Where provided, these should be in the most useful format, and they can be uploaded as "Source data" files linked to a main figure or table
- Include model definition files including the full list of parameters used
- Include code used for data analysis (e.g., R, MatLab)
- Avoid stating that data files are "available upon request"

Please indicate the figures or tables for which source data files have been provided:

We have provided our Matlab scripts for generating our figures on Github: https://github.com/PhantomSpike/DeReverb.<br>Our neural spiking data have been made freely available on Dryad: https://doi.org/10.5061/dryad.1c59zw3xv.
